# Five-factor model personality traits and cognitive function in five domains in older adulthood

**DOI:** 10.1186/s12877-019-1362-1

**Published:** 2019-12-05

**Authors:** Angelina R. Sutin, Yannick Stephan, Martina Luchetti, Antonio Terracciano

**Affiliations:** 1 Florida State University College of Medicine, 1115 W. Call Street, Tallahassee, FL USA; 2 Euromov, University of Montpellier, Montpellier, France

**Keywords:** Personality, Conscientiousness, Cognitive function, HCAP

## Abstract

**Background:**

Five-factor model (FFM) personality traits have been associated consistently with risk of Alzheimer’s disease and related dementias (ADRD). Less is known about how these traits are associated with functioning in specific domains of cognitive function in older adulthood.

**Methods:**

Participants (*N* = 2865) were drawn from the 2016 Harmonized Cognitive Assessment Protocol sub-study of the Health and Retirement Study (HRS). Participants completed a battery of cognitive tasks that measured performance in five domains: Memory (eight tasks), speed-attention-executive (five tasks), visuospatial ability (three tasks), fluency (one task), and numeric reasoning (one task). Participants completed an FFM personality measure as part of the regular HRS assessment in either 2014 or 2016. Linear regression was used to examine the association between the traits and each cognitive task and composite scores for the five domains, controlling for age, sex, race, ethnicity, and education. We also tested whether the associations were moderated by these sociodemographic factors or mental status.

**Results:**

Neuroticism was associated with worse performance on all of the cognitive tasks. Conscientiousness was associated with better performance across all five cognitive domains, although not necessarily with every task. Openness and Agreeableness were associated with better performance in all domains, except for numeric reasoning. Extraversion was associated with better speed-attention-executive and fluency. There was no robust evidence that the association between personality and cognition was moderated by sociodemographic characteristics or global cognitive function.

**Conclusions:**

Personality traits have pervasive associations with functioning across five cognitive domains. Consistent with the literature on personality and risk of ADRD, Neuroticism and Conscientiousness were associated with cognitive performance in the expected direction in all domains. Extraversion was the only trait that showed domain-specific associations. The present research supports models of personality and health in the context of cognition and suggests that personality is associated with intermediate markers of cognitive health.

## Background

Five Factor Model (FFM) personality traits [1] are associated consistently with significant cognitive impairment. In particular, individuals who score higher in Neuroticism (the tendency to experience negative emotions and stress) or lower in Conscientiousness (the tendency to be organized, disciplined, and responsible) are at greater risk of both mild cognitive impairments [2] and of Alzheimer’s disease [3]. Much of the work on personality and cognition in older adulthood has focused on global cognitive outcomes. And yet there are many cognitive domains that contribute to overall cognitive health [4]. The present research addresses five common domains of function [5]: Episodic memory, speed-attention-executive, visuospatial ability, fluency, and numeric reasoning. Episodic memory is memory for specific events in time and place. It includes memory for personal events that range from the distant past to events that just happened in the current moment. It is often measured with word lists that participants recall immediately and after a short delay. Speed and attention are functions that assess how quickly someone can respond to a stimulus (speed) and how well they can attend to the stimulus (attention); executive function includes these basic functions as well as cognitive flexibility. Visuospatial ability is the ability to visualize, rotate, and manipulate shapes in more than one dimension. Fluency is the ability to produce and use words correctly. Finally, numeric reasoning is the ability to manipulate numbers and includes basic arithmetic.

There are a number of reasons why personality traits may be associated with performance on cognitive tasks. Individuals who score higher in Neuroticism, for example, tend to be anxious and vulnerable to stress [6] and have self-presentational concerns around other people [7]. Such anxiety and self-conscientiousness are likely to inhibit performance on tasks administered in the presence of a tester. And indeed, individuals higher in Neuroticism tend to remember fewer words [8], respond slower on response time tasks [9, 10], have worse visuospatial performance [9], and produce fewer words on fluency tasks [11]. The associations between Extraversion (the tendency to be outgoing, sociable, and active) and cognition, in contrast, appear to be more domain specific. Extraversion has been associated with faster performance [10] and greater verbal fluency [11], whereas the association with episodic memory is more mixed [8–10], and it tends to be unrelated to visuospatial ability and numeric reasoning. These domain-specific associations are consistent with characteristics of this trait that include talkativeness [12] and vigor [13]. Cognitive flexibility and verbal abilities are core characteristics of Openness [14]. And, as expected, this trait tends to be related to better performance on tasks that include a verbal component [15] and on tasks that require cognitive flexibility [16, 17]. Agreeableness (the tendency to be trusting and empathetic) is sometimes associated with greater dementia risk [18] but is not associated consistently with performance on cognitive tasks [19]. Finally, Conscientiousness is associated with achievement striving and organization and a lifestyle that supports maintaining cognitive health across adulthood [20]. As such, it tends to be associated with better performance on a range of tasks [8, 11], but the associations are not always consistent [19]. For example, some find positive associations between Conscientiousness and better performance in tasks that measure speed, attention, and executive function [9] and others find no relation [10, 17, 21]. These differences may be due, in part, to differences in sample size (effects are generally modest and require large sample sizes for adequate power), differences in sample population (e.g., older versus younger adults; clinical versus nonclinical populations), and/or differences in measurement of both personality and tasks that measure speed, attention, and executive function. More broadly, and for these reasons, it is challenging to synthesize the literature on personality and measures of performance in these five cognitive domains. Focusing on large sample studies (*N* > 1000) that include validated measures of personality and cognition, the strongest evidence that personality is associated with specific cognitive domains is for the domains of memory and verbal fluency. In large samples of older adults, for example, higher Neuroticism tends to be associated with worse memory performance, whereas higher Openness and Conscientiousness tend to be associated with better memory [8, 22]. Further, our recent meta-analysis on personality and verbal fluency (meta-analytic *N* > 85,000) indicated that higher Neuroticism was associated with lower fluency whereas higher Extraversion, Openness, and Conscientiousness were associated with greater fluency [11]. As such, stronger hypotheses can be made for memory and fluency based on this previous literature than for speed-attention-executive, visuospatial ability, and numeric reasoning.

The literature on personality and cognition suggests that the traits may have differential associations with different aspects of cognitive function. Previous research on personality and cognition has tended to focus on one or two personality traits (typically Neuroticism and Extraversion) and/or individual cognitive functions (e.g., episodic memory). Although there is growing recognition of the importance of including all five traits and tasks from multiple cognitive domains, such studies remain relatively rare. The present research sought to unify the literature by examining the association between all FFM traits and five common domains of cognitive function in a relatively large sample of older adults. The large sample also allowed us to examine whether the associations varied by sociodemographic characteristics. Based on the literature on personality and cognition, we expected higher Neuroticism to be associated with worse performance on all of the cognitive tasks and higher Conscientiousness and Openness to be associated with better performance. In contrast, we expected higher Extraversion to be associated with better performance on speed and fluency tasks. We did not expect Agreeableness to be associated with the cognitive tasks. In addition to the main effect, we examine whether the association between personality and cognition is moderated by age, sex, race, ethnicity, education, or global cognitive function.

## Method

### Participants and procedure

Participants were part of the 2016 Harmonized Cognitive Assessment Protocol (HCAP), an ongoing sub-study of the Health and Retirement Study (HRS). Participants were selected to be a part of the HCAP assessment if they were 65 years or older and completed the 2016 interview of the HRS. Of the eligible participants, a subset was randomly selected and invited to participate in the HCAP assessment (*N* = 5500). A total of 3496 participants completed at least some part of it. Participants could have the HCAP assessment administered in either English or Spanish. Participants who also completed the personality measure in either the 2014 or 2016 regular HRS assessment were selected for analysis. A total of 2865 participants had complete data on personality and some measures in HCAP. The sample was on average 76.49 (SD = 7.36) years old, with 66% of the sample between the ages of 65–79, 30% between the ages of 80–89, and 4% over the age of 90. The sample was 60% female, 14% African American, 9% Hispanic, and had an average of 12.93 (SD = 2.98) years of education. By comparison, the Census estimates that the US population aged 65 and older is 56% female, 9% African American, 8% Hispanic, and that 84% of this population had completed high school [23].

Analytic samples ranged from 2456 (numeric reasoning) to 2814 (CERAD immediate recall, verbal fluency) based on missing data across the cognitive tasks. Compared to participants who had the personality assessment available, participants without the personality assessment and thus not included in the analyses (*n* = 629) were older (*d* = .24, *p* < .01), more likely to be Hispanic (χ^2^ = 38.16, *p* < .01), more likely to be a race other than white (χ^2^ = 59.85, *p* < .01), and had fewer years of education (*d* = .38, *p* < .01); there was no difference in participant sex. Further, there were differences on all of the cognitive tasks, with *d*s that ranged from .30 (*p* < .01; Backward Count) to .71 (*p* < .01; Mini Mental State Examination); across all cognitive tasks, participants who also had the personality assessment performed better than participants who did not have personality. More information on the HCAP assessment, sampling, and how to obtain the data can be found at https://hrs.isr.umich.edu/news/2016-harmonized-cognitive-assessment-protocol-hcap-early-version-10. The Health and Retirement Study make their data available to the public, but access to the HCAP data requires an additional authentication process to verify the identity of the person and institute requesting access to the data. We acquired this administrative permission to access the HCAP data. Information about how to access the HCAP data can be found at https://hrs.isr.umich.edu/data-products/cognition-data. The Institutional Review Board at the Florida State University approved this research (protocol #IRB00000446, “Secondary Data Analysis of Public Health Databases”).

### Measures

#### Personality

Participants completed the Midlife Development Inventory (MIDI [24]) as part of the Leave-Behind Questionnaire in either 2014 or 2016. The MIDI assesses FFM personality traits with 26 adjectives. Items on the MIDI measured Neuroticism (e.g., moody; alpha = .71), Extraversion (e.g., talkative; alpha = .75), Openness (e.g., creative; alpha = .80), Agreeableness (e.g., helpful; alpha = .79), and Conscientiousness (e.g., organized; alpha = .71). Items were rated on a scale from 1 (a lot) to 4 (not at all) and reverse scored in the direction of the trait label. The mean was taken across items for each trait (range 1–4).

#### Cognition

HCAP is an extensive assessment of cognitive function that covers the domains of episodic memory, speed-attention-executive, visuospatial ability, fluency, and numeric reasoning. Detailed information about test administration and scoring can be found in Weir and colleagues [25].

#### Episodic memory

Participants completed several measures of episodic memory, measured as immediate recall, delayed recall, and recognition. Participants completed the CERAD Word List Learning and Recall Task. Participants were presented visually with a list of 10 words, two seconds at a time for each word. Participants read each word and after the last word were asked to recall as many words from the list as possible (immediate recall). After a short delay, participants were again asked to recall as many words from the list as possible (delayed recall). Finally, participants were shown 10 target words and 10 foils and were asked to indicate which words were on the original list (recognition). Participants also completed two story memory tasks: Brave Man and the Wechsler Memory Scale Logical Memory I. Both tasks involved being read a passage and being asked to report back the main points of the story immediately and after a short delay. The Logical Memory task also included a recognition test, in which participants were asked 15 yes/no questions about the story.

#### Speed-attention-executive

This domain was assessed with several tasks. In the Letter Cancellation Test, participants were given one minute to cross out as many “P” and “W” letters as possible from a large grid of letters. The score was the last letter gotten to at the one-minute mark. Participants completed the Backward Count task as a measure of processing speed in which they counted backward from 100 as fast as possible. The count of numbers said in 30 s was the score. The Symbol-Digit Modalities Test had random geometric figures and a separate key that paired numbers with each figure. Participants were asked to substitute a number for each figure on the sheet of paper. The score was the number of correct pairings made in 90 s. The Trail Making Test had two parts. Part A was a sheet of numbers in circles on a page and participants were asked to connect the consecutively numbered circles as fast as possible. Trails B included letters as well as numbers and participants had to switch between numbers and letters as quickly as possible. For both parts, the outcome was time to complete the task (i.e., slower times indicated worse performance).

#### Visuospatial ability

The CERAD Constructional Praxis task required participants to copy geometric forms that varied in difficulty both immediately and after a short delay. Note that performance on the delay task reflects visual memory as well as visuospatial ability. Raven’s Standard Progressive Matrices were geometric pictures with a small section missing. Participants were asked to choose the correct picture from a set that correctly completed the picture. Participants completed 17 pictures from the total Raven’s test.

#### Fluency

Fluency was measured with a semantic verbal fluency task. Specifically, participants were asked to name as many animals as possible in 60 s.

#### Numeric reasoning

The HRS number series was a measure of numeric reasoning. Participants were presented with a series of numbers with one or two numbers missing. Participants were asked to identify the missing numbers. The test was not timed and participants could take as much time as necessary to complete it. The test was adaptive, such that items got more or less difficult depending on participants’ responses.

#### Global cognitive function

Finally, participants completed the Mini-Mental State Examination (MMSE) as a measure of global cognitive function [26]. The MMSE was used to test cognitive status as a moderator of the relation between personality and the cognitive tasks and not as an outcome. The total MMSE score was dichotomized into no impairment (≥24, coded as 0) and any impairment (≤23, coded as 1).

#### Covariates

Covariates were self-reported age in years, sex (female = 1, male = 0), race (African American = 1 [dummy variable 1], other/unknown = 1 [dummy variable 2] both compared to white = 0], Hispanic ethnicity (1 = yes, 0 = no), and education in years. Some participants chose to have the HCAP administered in Spanish. We included language of administration (0 = English, 1 = Spanish) as a covariate.

### Statistical approach

We used linear regression to examine the association between personality and the cognitive measures. Specifically, each cognitive task was predicted separately by each trait, controlling for age, sex, race, ethnicity, education, and language of HCAP administration. For the domains with multiple tasks (episodic memory, speed-attention-executive, visuospatial ability), in addition to the individual tasks, the score for each task was standardized and then the mean taken across the tasks within the domain as an overall measure of the domain (Trails A and Trails B were multiplied by − 1 to reverse the direction of the scoring to be consistent with the scoring of the other tasks in this domain). We then tested whether the association between personality and each domain was moderated by age, sex, race, ethnicity, education, or mental status. Finally, we did a threshold analysis to test whether the traits were associated with performance below a threshold. Specifically, we tested whether personality was associated with risk of performing at least one standard deviation below the mean (a relatively arbitrary but standard cutoff for threshold analyses) for each of the five domains. For all analyses, *p* was set to <.01, two-tailed.

## Results

Descriptive statistics for all study variables are shown in Table 1. Bivariate correlations between the traits and the cognitive tasks are shown in Additional file 1: Tables S1, S2, and S3. Although there were some minor violations of the assumptions of linear regression, the bivariate correlations were similar across the Pearson correlations (parametric) and Spearman correlations (nonparametric) that suggest the associations found in the regression analyses are not simply due to violations of the assumptions of linear regression. Tables 2, 3, and 4 show the association between personality and performance across the five domains of cognitive function. Note that for all analyses, the pattern of associations and significance was identical when outliers were removed from the analysis. Several patterns emerged from the analyses that were consistent with our hypotheses. First, Neuroticism was associated with worse performance on every cognitive task: Individuals higher in Neuroticism performed worse on measures of episodic memory, speed-attention-executive, visuospatial ability, fluency, and numeric reasoning. Second, although not apparent across all individual tasks, higher Openness and higher Conscientiousness were both associated generally with better performance. Third, the association between Extraversion and cognition was limited to better performance in the speed-attention-executive and fluency domains; Extraversion was unrelated to episodic memory, visuospatial ability, and numeric reasoning. Fourth, although unexpected, Agreeableness was associated with better performance in four out of the five domains (all domains except numeric reasoning). Fifth, the personality traits tended to have more associations with the speed-attention-executive and fluency tasks than the other cognitive functions. Within the memory domain, the traits had more associations with the word list learning and recall tasks than with the story memory tasks. Finally, as could be expected, the associations were slightly stronger with the scores combined across tasks, and the effect sizes were generally small. For episodic memory, the adjusted R^2^ was .278 for the covariates only model and change in adjusted R^2^ ranged from .004 (Agreeableness and Conscientiousness) to .009 (Neuroticism) for personality. For speed-attention-executive, the adjusted R^2^ was .391 for the covariates only model and change in adjusted R^2^ ranged from .005 (Extraversion) to .015 (Conscientiousness) for personality. For visuospatial ability, the adjusted R^2^ was .313 for the covariates only model and change in adjusted R^2^ ranged from .000 (Extraversion) to .011 (Openness) for personality. For fluency, the adjusted R^2^ was .205 for the covariates only model and change in adjusted R^2^ ranged from .003 (Neuroticism, Extraversion, and Conscientiousness) to .011 (Openness) for personality. For numeric reasoning, the adjusted R^2^ was .292 for the covariates only model and change in adjusted R^2^ ranged from .000 (Extraversion) to .005 (Neuroticism) for personality. See Additional file 1: Tables S4, S5, and S6 for full reporting of the adjusted R^2^ for each analysis.

**Table 1:** Descriptive Statistics for All Study Variables

Variable	Mean (SD); range or %
Age (years)	76.49 (7.36); 65–99
Sex (female)	60%
Race (African American)	14%
Race (Other)	4%
Race (white)	82%
Hispanic (yes)	9%
Education (years)	12.93 (2.98); 0–17
Language of HCAP administration (Spanish)	4%
Mini-Mental State Examination	27.13 (3.26); 1–30
Personality	
Neuroticism	1.91 (.58); 1–4
Extraversion	3.18 (.57); 1–4
Openness	2.87 (.57); 1–4
Agreeableness	3.50 (.51); 1–4
Conscientiousness	3.26 (.40); 1–4
Memory	
CERAD Immediate (*n* = 2816)	4.39 (1.77); 0-10^a^
CERAD Delayed (*n* = 2803)	5.33 (2.55); 0–10
CERAD Recognition (*n* = 2807)	9.03 (1.61); 0–10
Brave Man Immediate (*n* = 2807)	7.27 (2.32); 0–12
Brave Man Delayed (*n* = 2770)	5.31 (3.23); 0–12
Logical Memory Immediate (*n* = 2790)	10.20 (5.01); 0–23
Logical Memory Delayed (*n* = 2762)	7.73 (5.37); 0–25
Logical Memory Recognition (*n* = 2756)	10.47 (2.64); 0–15
Composite (*n* = 2817)	−.01 (.74)^b^
Speed-Attention-Executive	
Letter Cancellation (*n* = 2721)	20.28 (6.61); 0–65
Backward Count (*n* = 2786)	30.50 (10.71); 0–70
Symbol Digit (*n* = 2715)	33.73 (11.94); 0–71
Trails A (*n* = 2736)	51.66 (30.77); 3–300
Trails B (*n* = 2471)	123.62 (57.58); 32–300
Composite (*n* = 2808)	−.02 (.47)^b^
Visuospatial	
Constructional Praxis Immediate (*n* = 2796)	8.39 (2.22); 0–11
Constructional Praxis Delayed (*n* = 2792)	6.09 (3.12); 0–11
Raven Matrices (*n* = 2791)	12.73 (3.67); 0–17
Composite (*n* = 2808)	−.01 (.84)^b^
Verbal Fluency (*n* = 2816)	16.52 (6.39); 0–43
Number Series (*n* = 2457)	523.95 (30.44); 409–584

*Note*. *N* = 2865. The analytic *n*s vary for cognition due to missing data. ^a^ After the first trial, participants completed two additional trials, in which the same words were presented. Participants read the words again and were then asked to recall all words after each presentation. We follow the Weir et al. (2016)‘s guidelines and use the first trial as the measure of immediate recall. Participants overall did better on delayed recall because they practiced the same list three times. For this reason, the mean number of words recalled is higher for delayed than immediate recall. ^b^ Score is a composite of multiple tasks that were standardized before aggregating

**Table 2:** Associations Between Personality Traits and Episodic Memory

Trait	Episodic Memory								
	Combined	CERAD			Brave Man		Logical		
		Immediate	Delayed	Recognition	Immediate	Delayed	Immediate	Delayed	Recognition
Neuroticism	−.10*	−.08*	−.08*	−.07*	−.09*	−.05*	−.09*	−.07*	−.05*
Extraversion	.03	.03	.03	.02	.01	.00	.03	.01	.02
Openness	.07*	.08*	.06*	.01	.04	.04	.07*	.05*	.07*
Agreeableness	.06*	.06*	.04	.04	.04	.03	.04	.03	.06*
Conscientiousness	.06*	.07*	.06*	.05*	.03	.04	.04	.04	.03
Sample size	2817	2816	2803	2807	2807	2770	2790	2762	2756

*Note*. Coefficients are standardized beta coefficients from linear regression controlling for age, sex, race, ethnicity, education, and language of test administration

^*^p < .01

**Table 3:** Associations Between Personality Traits and Speed-Attention-Executive

Trait	Speed-Attention-Executive					
	Combined	Letter Cancelation	Backwards Count	Symbol Digit	Trails A^a^	Trails B^a^
Neuroticism	−.10*	−.08*	−.05*	−.09*	−.08*	−.08*
Extraversion	.07*	.07*	.07*	.06*	.04	.04
Openness	.08*	.07*	.05*	.08*	.07*	.06*
Agreeableness	.10*	.08*	.09*	.06*	.07*	.08*
Conscientiousness	.12*	.10*	.08*	.11*	.09*	.11*
Sample size	2808	2721	2786	2715	2736	2471

*Note*. Coefficients are standardized beta coefficients from linear regression controlling for age, sex, race, ethnicity, education, and language of test administration. ^a^ Trails A and B were multiplied by −1 to make the direction of the scoring consistent with the other tasks in this domain (i.e., higher scores indicate worse performance)

^*^p < .01

**Table 4:** Associations Between Personality Traits and Visuospatial Ability, Fluency, and Numeric Reasoning

Trait	Visuospatial Ability				Fluency	Numeric Reasoning
	Combined	Constructional Praxis		Pattern Reasoning		
		Immediate	Delayed	Raven Matrices	Semantic Fluency	Number Series
Neuroticism	−.10*	−.06*	−.09*	−.09*	−.06*	−.07*
Extraversion	.03	.01	.04	.02	.06*	.00
Openness	.11*	.09*	.08*	.10*	.11*	.04
Agreeableness	.08*	.06*	.07*	.06*	.08*	.02
Conscientiousness	.09*	.08*	.08*	.06*	.06*	.06*
Sample size	2808	2796	2792	2791	2816	2457

*Note*. Coefficients are standardized beta coefficients from linear regression controlling for age, sex, race, ethnicity, education, and language of test administration

^*^p < .01

There was little evidence that the associations between personality and the five cognitive domains were moderated by sociodemographic factors or mental status. There was a stronger association between Agreeableness and episodic memory at relatively lower than higher levels of education (β_interaction_ = −.04, *p* < .01), whereas higher education amplified the effect of Openness on fluency (β_interaction_ = .04, *p* < .01). There was also a stronger association between Neuroticism and visuospatial abilities at relatively older than younger ages (β_interaction_ = −.05, *p* < .01). Finally, although apparent across race, the association between Conscientiousness and visuospatial abilities was stronger among African American participants than white participants (β_interaction_ = .06, *p* < .01). None of the associations was moderated by sex, ethnicity, or global cognitive function. Further, the associations were similar when participants with cognitive impairment as indicated by the MMSE (*n* = 277) were excluded from the analysis.

Finally, the threshold analyses generally paralleled the linear regressions (Table 5). Specifically, higher Neuroticism was associated with a 20% (fluency) to 40% (speed-attention-executive) increased risk of poor performance, whereas lower Conscientiousness was associated with a 19% (fluency) to 39% (speed-attention-executive) increased risk. Extraversion was likewise associated with 16% (visuospatial ability) to 20% (speed-attention-executive and fluency) greater likelihood of better performance, Openness was associated with a 19% (episodic memory) to 35% (visuospatial ability) greater likelihood of better performance, and Agreeableness was associated with a 19% (visuospatial ability) to 33% (speed-attention-executive) greater likelihood of better performance. Of note, with the exception of Openness, the strongest associations in the threshold analyses were for the speed-attention-executive domain.

**Table 5:** Associations Between Personality Traits and Risk of Performance One Standard Deviation Below the Mean

Trait	Episodic Memory	Speed-Attention-Executive	Visual-Spatial Ability	Fluency	Numeric Reasoning
Neuroticism	1.22 (1.08–1.36)*	1.40 (1.25–1.57)*	1.25 (1.12–1.40)*	1.20 (1.08–1.34)*	1.27 (1.12–1.45)*
Extraversion	.89 (.80–.99)	.83 (.75–.93)*	.85 (.76–.95)*	.83 (.75–.92)*	.88 (.78–1.01)
Openness	.84 (.75–.94)*	.81 (.72–.90)*	.74 (.66–.83)*	.81 (.73–.90)*	.80 (.70–.91)*
Agreeableness	.80 (.72–.89)*	.75 (.67–.83)*	.84 (.75–.93)*	.84 (.76–.93)*	.79 (.69–.91)*
Conscientiousness	.78 (.70–.88)*	.72 (.65–.81)*	.81 (.72–.90)*	.85 (.76–.94)*	.81 (.71–.93)*
Sample size	2817	2808	2808	2816	2457

*Note*. Coefficients are odds ratios (95% Confidence Interval) from logistic regression controlling for age, sex, race, ethnicity. Education, and language of test administration

^*^p < .01

## Discussion

The present research used a large sample of older adults, a measure of all five FFM traits, and tasks that tap into five core domains of cognition to examine the relation between personality and specific cognitive functions. Consistent with the literature on personality and dementia risk [18], higher Neuroticism and lower Conscientiousness were associated with worse performance across the five domains. Openness likewise emerged as a broad correlate of better cognitive function, whereas the associations were more domain-specific for Extraversion. Surprisingly, Agreeableness was associated with better performance across most of the domains.

Several shared mechanisms are likely to contribute to the personality and cognition associations, but there are specific mechanisms that are potentially more relevant for some traits than others. For example, individuals higher in Neuroticism are prone to anxiety [6] and tend to perform worse in the presence of other people [7]. Such self-consciousness and performance anxiety likely inhibit the ability to perform well, especially in front of a tester. Neuroticism is also associated with a number of risk factors for dementia that may contribute to worse performance on specific cognitive tests. Individuals higher in Neuroticism are more likely to be sedentary and less likely to engage in physical activity [27]. They are also more likely to smoke [28] and experience depression [29]. A physiological pathway is also possible. The stress hormones (e.g., cortisol [30]), neurotrophic factors (e.g., BDNF [31]), and systemic inflammation [32] associated with Neuroticism may also contribute to worse performance.

Achievement striving and industriousness are core components of Conscientiousness [6], and this tendency for working hard to be successful likely extends to cognitive tasks in at least two ways. First, this striving may help individuals develop skills and strategies that facilitate performance, especially for difficult tasks. Second, it may be motivation to try as hard as possible to perform well while completing the tasks. In addition, like Neuroticism, there are behavioral and physiological pathways that likely support better performance. Individuals higher in Conscientiousness engage in more frequent physical activity and tend not to be sedentary [27], they are less likely to smoke [28], and more likely to have healthier weight across adulthood [33]. These healthier behaviors tend to protect cognitive function [34]. Conscientiousness is also associated with healthier cardiometabolic [35] and inflammatory [32] profiles that may likewise serve to preserve cognition.

Openness traditionally has been associated with better performance on cognitive tasks. Of the five traits, it is the only one that has cognitive characteristics as a core component. That is, part of the definition of Openness is cognitive flexibility and engagement [14]. This trait also has the strongest and most consistent associations with education: Individuals higher in Openness tend to achieve more years of education [36]. It is of note, then, that the associations across the five cognitive domains in this research emerged independent of education. If education is not considered a confounding factor (i.e., openness is a determinant of educational achievement more than a byproduct), the associations between openness and cognition would generally have effect sizes that are twice as large. Like Neuroticism and Conscientiousness, there may be behavioral factors that mediate the relation between Openness and better cognitive performance in older adulthood. For example, individuals higher in Openness tend to eat healthier diets [37] and are more physically active [38]. They also tend to engage in more cognitively demanding activities across the lifespan [39]. The end result may be preserved cognitive function in older adulthood.

As expected, the association between Extraversion and cognition was limited to specific domains of function. Specifically, Extraversion was associated with better performance on tests of speed-attention-executive and fluency. These associations are likely a reflection of some of the core aspects of this trait. Individuals higher in Extraversion tend to talk more [12] and have more vigor [13]. Such characteristics translate into a better ability to produce words from a specific category and faster reactions times. Previous research has suggested that Extraversion tends to be unrelated to episodic memory [9, 17]. We likewise found no association with memory, and that Extraversion was also unrelated to both visuospatial and numeric abilities. These domain-specific associations are consistent with the literature on Extraversion and cognitive impairment that indicates that Extraversion is unrelated to dementia risk [18]. It suggests that the cognitive benefits associated with Extraversion are domain specific rather than generally protective of global cognition.

Surprisingly, Agreeableness was associated with four out of the five cognitive domains. When combined across multiple samples, there is some evidence for a small protective effect of Agreeableness on dementia risk [18]. Still, it tends to be unrelated to specific cognitive functions [9, 17]. Thus, the fairly consistent positive associations across the tasks was unexpected. Individuals higher in Agreeableness tend to volunteer, especially in a critical time before the transition to older adulthood [40]. Given that volunteering has been found to help maintain cognitive function [41], it may be one mechanism that contributes to the association between Agreeableness and better cognitive function in older adulthood. This pattern of associations, however, should be interpreted with caution until it is replicated.

It is of note that across the five domains, personality had the most associations with performance in the domain that measured speed-attention-executive. This pattern is consistent with research in other domains that indicates that individual differences in personality show stronger associations when more effort is needed to complete the task [38]. For example, personality is unrelated to resting metabolic rate but the expected associations are apparent when participants are asked to walk as fast as possible [42]. This pattern suggests that the characteristics of the traits that contribute to better performance are more engaged when there is a time component, and thus greater urgency, for performance.

Across traits and cognitive domains, the association between personality and cognitive performance was relatively small, with most associations ≤ .10. Given that cognitive performance is determined by factors that range from genetic [43] to environmental [44], it is not surprising that any individual factor, such as personality, would have a small association. It is of note, however, despite the small magnitude, the pattern of associations within and across cognitive domains was similar and consistent with the growing literature on personality and cognition [8, 11, 19, 22].

There was little evidence that the associations were moderated by sociodemographic characteristics or mental status. Even when moderation was found, the interactions indicated that the associations were slightly stronger in one group compared to the other. Overall, the lack of moderation indicates that the relation between personality and cognition is similar across socio-demographic groups (e.g., women and men, low and high education) and not dependent on overall global cognition. The latter finding is particularly noteworthy because personality traits maintain predictive power despite deterioration of cognitive abilities.

Lifespan models of personality and health posit that personality contributes to significant health outcomes across the lifespan through multiple mechanisms, including behavioral and physiological pathways [20]. Such models are increasingly being applied to cognitive outcomes to better understand how personality contributes to risk of significant cognitive impairment in older adulthood [45, 46]. Within this context, the current research suggests that personality is also associated with intermediate markers of cognitive health in older adulthood that should be considered in the pathway to significant impairment. As such, the present research broadens lifespan models to create a more detailed approach to personality and cognition and begins to place cognitive performance in specific domains in the pathway from personality to significant cognitive outcomes.

This research had several strengths, including a relatively large sample of older adults, a validated FFM measure of personality traits, and standard tasks that measured five domains of cognitive function. This research also had several limitations. The speed-attention-executive domain, for example, could be considered as three separate domains instead of one. The tasks were combined under one domain because many of the tasks tapped in to multiple functions and thus could not differentiate between them. The associations at the task-level support the decision to collapse across the three functions (i.e., the results were similar across the tasks within the domain). Still, in future work it would be worthwhile to have multiple tasks that measure each of these components. In addition, we only had one measure of executive function (Trails B). Executive function is thought to be the integration of multiple functions (e.g., inhibitory control, working memory, cognitive flexibility [47]) but we could not address these individual functions in the present research. Finally, the data were cross-sectional and thus unable to speak to the temporal ordering of the relations. For example, severe cognitive impairment is associated with change in personality [48], although not prior to the onset of impairment [49]. In future research, it would be important to have longitudinal assessments of both personality and cognition to examine their interrelations over time. Despite these limitations, the present research is a step toward better understanding the relation between personality and specific cognitive functions in older adulthood.

## Conclusions

The present research indicates that FFM personality traits have differential associations with five domains of cognitive function. The findings show that traits that are associated consistently with dementia risk (Neuroticism, Conscientiousness) are also associated with intermediate markers of cognitive function. Such findings demonstrate the role of individual differences in psychological functioning in cognitive health in older adulthood.

## Supplementary information

**Additional file 1: Table S1.** Pearson and Spearman Correlations Between Personality and Episodic Memory. **Table S2.** Pearson and Spearman Correlations Between Personality and Speed-Attention-Executive. **Table S3.** Pearson and Spearman Correlations Between Personality and Visuospatial Ability, Fluency, and Numeric Reasoning. **Table S4.** Adjusted R^2^ for each Regression Analysis Predicting Episodic Memory from Personality and the Covariates.**Table S5.** Adjusted R^2^ for each Regression Analysis Predicting Speed-Attention-Executive from Personality and the Covariates. **Table S6.** Adjusted R^2^ for each Regression Analysis Predicting Visuospatial Ability, Fluency, and Numeric Reasoning from Personality and the Covariates.

## Data Availability

The datasets supporting the conclusions of this article are available for public download from HRS: http://hrsonline.isr.umich.edu/

## Abbreviations

FFM: Five Factor Model
HCAP: Harmonized Cognitive Assessment Protocol
HRS: Health and Retirement Study
MMSE: Mini-Mental State Examination

## References

[1] McCrae RR, John OP (1992). An introduction to the five-factor model and its applications. J Pers.

[2] Terracciano A, Stephan Y, Luchetti M, Albanese E, Sutin AR (2017). Personality traits and risk of cognitive impairment and dementia. J Psychiatr Res.

[3] Duberstein PR, Chapman BP, Tindle HA, Sink KM, Bamonti P, Robbins J, Jerant AF, Franks P (2011). Personality and risk for Alzheimer’s disease in adults 72 years of age and older: a 6-year follow-up. Psychol Aging.

[4] Bäckman L, Jones S, Berger AK, Laukka EJ, Small BJ (2005). Cognitive impairment in preclinical Alzheimer’s disease: a meta-analysis. Neuropsychology.

[5] Lezak MD (2004). Neuropsychological assessment.

[6] Costa PT (1992). McCrae RR: revised NEO personality inventory (NEO-PI-R) and the NEO five-factor inventory (NEO-FFI) professional manual.

[7] Eldesouky L, English T (2018). Individual differences in emotion regulation goals: does personality predict the reasons why people regulate their emotions?. J Personal.

[8] Luchetti M, Terracciano A, Stephan Y, Sutin AR (2016). Personality and cognitive decline in older adults: data from a longitudinal sample and meta-analysis. J Gerontol B Psychol Sci Soc Sci.

[9] Chapman BP, Benedict RH, Lin F, Roy S, Federoff HJ, Mapstone M (2017). Personality and performance in specific neurocognitive domains among older persons. Am J Geriatr Psychiatry.

[10] Wettstein M, Tauber B, Kuźma E, Wahl HW (2017). The interplay between personality and cognitive ability across 12 years in middle and late adulthood: evidence for reciprocal associations. Psychol Aging.

[11] Sutin AR, Stephan Y, Damian RI, Luchetti M, Strickhouser JE, Terracciano A (2019). Five-factor model personality traits and verbal fluency in 10 cohorts. Psychol Aging.

[12] Mehl MR, Gosling SD, Pennebaker JW (2006). Personality in its natural habitat: manifestations and implicit folk theories of personality in daily life. J Pers Soc Psycho.

[13] Armon G, Shirom A (2011). The across-time associations of the five-factor model of personality with vigor and its facets using the bifactor model. J Pers Assess.

[14] McCrae RR, Costa PT, Hogan R, Johnson JA, Briggs SR (1997). Conceptions and correlates of openness to experience. Handbook of personality psychology.

[15] Sharp ES, Reynolds CA, Pedersen NL, Gatz M (2010). Cognitive engagement and cognitive aging: is openness protective?. Psychol Aging.

[16] DeYoung CG, Peterson JB, Higgins DM (2005). Sources of openness/intellect: cognitive and neuropsychological correlates of the fifth factor of personality. J Pers.

[17] Weinstein G, Elran Barak R, Schnaider Beeri M, Ravona-Springer R (2018). Personality traits and cognitive function in old-adults with type-2 diabetes. Aging Ment Health.

[18] Terracciano A, Sutin AR, An Y, O’Brien RJ, Ferrucci L, Zonderman AB, Resnick SM (2014). Personality and risk of Alzheimer’s disease: new data and meta-analysis. Alzheimers Dement.

[19] Curtis RG, Windsor TD, Soubelet A (2015). The relationship between Big-5 personality traits and cognitive ability in older adults - a review. Neuropsychol Dev Cogn B Aging Neuropsychol Cogn.

[20] Friedman HS, Kern ML, Hampson SE, Duckworth AL (2014). A new life-span approach to conscientiousness and health: combining the pieces of the causal puzzle. Dev Psychol.

[21] Graham EK, Lachman ME (2014). Personality traits, facets and cognitive performance: age differences in their relations. Personal Individ Differ.

[22] Allen MS, Laborde S, Walter EE (2019). Health-related behavior mediates the association between personality and memory performance in older adults. J Appl Gerontol.

[23] Roberts AW, Ogunwole SU, Rabe MA. The population 65 years and older in the United States: 2016. In: US Census Bureau, ACS-38 edn. Washington, DC; 2018.

[24] Lachman ME, Weaver SL. Midlife development inventory (MIDI) personality scales: scale construction and scoring. Unpublished Technical Report. In*.* Brandeis University; 1997.

[25] Weir DR, Langa KM, Ryan LH. Harmonized Cognitive Assessment Protocol (HCAP): study protocol summary; 2016.

[26] Folstein MF, Folstein SE, Fanjiang G. Mini-mental state examination: clinical guide and User’s guide. Lutz; 2001.

[27] Sutin AR, Stephan Y, Luchetti M, Artese A, Oshio A, Terracciano A (2016). The five factor model of personality and physical inactivity: a meta-analysis of 16 samples. J Res Pers.

[28] Hakulinen C, Hintsanen M, Munafò MR, Virtanen M, Kivimäki M, Batty GD, Jokela M (2015). Personality and smoking: individual-participant meta-analysis of nine cohort studies. Addiction.

[29] Kendler KS, Myers J (2010). The genetic and environmental relationship between major depression and the five-factor model of personality. Psychol Med.

[30] Nater UM, Hoppmann C, Klumb PL (2010). Neuroticism and conscientiousness are associated with cortisol diurnal profiles in adults--role of positive and negative affect. Psychoneuroendocrinology.

[31] Terracciano A, Lobina M, Piras MG, Mulas A, Cannas A, Meirelles O, Sutin AR, Zonderman AB, Uda M, Crisponi L (2011). Neuroticism, depressive symptoms, and serum BDNF. Psychosom Med.

[32] Luchetti M, Barkley JM, Stephan Y, Terracciano A, Sutin AR (2014). Five-factor model personality traits and inflammatory markers: new data and a meta-analysis. Psychoneuroendocrinology.

[33] Sutin AR, Boutelle K, Czajkowski SM, Epel ES, Green PA, Hunter CM, Rice EL, Williams DM, Young-Hyman D, Rothman AJ (2018). Accumulating Data to Optimally Predict Obesity Treatment (ADOPT) Core measures: psychosocial domain. Obesity (Silver Spring).

[34] Norton S, Matthews FE, Barnes DE, Yaffe K, Brayne C (2014). Potential for primary prevention of Alzheimer’s disease: an analysis of population-based data. Lancet Neurol.

[35] Sutin AR, Costa PT, Uda M, Ferrucci L, Schlessinger D, Terracciano A (2010). Personality and metabolic syndrome. Age.

[36] Sutin AR, Stephan Y, Luchetti M, Robins RW, Terracciano A (2017). Parental educational attainment and adult offspring personality: An intergenerational lifespan approach to the origin of adult personality traits. J Pers Soc Psychol.

[37] Mõttus R, Realo A, Allik J, Deary IJ, Esko T, Metspalu A (2012). Personality traits and eating habits in a large sample of Estonians. Health Psychol.

[38] Wilson KE, Dishman RK (2015). Personality and physical activity: a systematic review and meta-analysis. Personal Individ Differ.

[39] Stephan Y, Boiché J, Canada B, Terracciano A (2014). Association of personality with physical, social, and mental activities across the lifespan: Findings from US and French samples. Br J Psychol.

[40] King HR, Jackson JJ, Morrow-Howell N, Oltmanns TF (2015). Personality accounts for the connection between volunteering and health. J Gerontol B Psychol Sci Soc Sci.

[41] Infurna FJ, Okun MA, Grimm KJ (2016). Volunteering is associated with lower risk of cognitive impairment. J Am Geriatr Soc.

[42] Terracciano A, Schrack JA, Sutin AR, Chan W, Simonsick EM, Ferrucci L (2013). Personality, metabolic rate and aerobic capacity. PLoS One.

[43] Savage JE, Jansen PR, Stringer S, Watanabe K, Bryois J, de Leeuw CA, Nagel M, Awasthi S, Barr PB, Coleman JRI (2018). Genome-wide association meta-analysis in 269,867 individuals identifies new genetic and functional links to intelligence. Nat Genet.

[44] Zhang X, Chen X (2018). The impact of exposure to air pollution on cognitive performance. Proc Natl Acad Sci U S A.

[45] Segerstrom SC. Personality and incident Alzheimer’s disease: theory, evidence, and future directions. J Gerontol B Psychol Sci Soc Sci. 2018; 10.1093/geronb/gby063 PMC7768711 29846724

[46] Sutin AR, Stephan Y, Aschwanden D, Luchetti M, Strickhouser JE, Terracciano A. Evaluations of a previous day as a pathway between personality and healthy cognitive aging. J Aging Health. 2019; 10.1177/0898264319843451. 10.1177/0898264319843451 PMC7011188 31030604

[47] Diamond A (2013). Executive functions. Annu Rev Psychol.

[48] Islam M, Mazumder M, Schwabe-Warf D, Stephan Y, Sutin AR, Terracciano A (2018). Personality changes with dementia from the informant perspective: new data and meta-analysis. JAMDA.

[49] Terracciano A, An Y, Sutin AR, Thambisetty M, Resnick SM (2017). Personality change in the preclinical phase of Alzheimer disease. JAMA Psychiatry.

