# Supplementary material for: Five-factor model personality traits and cognitive function in five domains in older adulthood
Source: BMC Geriatr. 2019 Dec 5;19:343. doi: 10.1186/s12877-019-1362-1 (PMC6896269; doi:10.1186/s12877-019-1362-1)
Supplement: Supplementary file 1 — Additional file 1: Table S1. Pearson and Spearman Correlations Between Personality and Episodic Memory. Table S2. Pearson and Spearman Correlations Between Personality and Speed-Attention-Executive. Table S3. Pearson and Spearman Correlations Between Personality and Visuospatial Ability, Fluency, and Numeric Reasoning. Table S4. Adjusted R2 for each Regression Analysis Predicting Episodic Memory from Personality and the Covariates.Table S5. Adjusted R2 for each Regression Analysis Predicting Speed-Attention-Executive from Personality and the Covariates. Table S6. Adjusted R2 for each Regression Analysis Predicting Visuospatial Ability, Fluency, and Numeric Reasoning from Personality and the Covariates. [file 12877_2019_1362_MOESM1_ESM.docx]

Supplemental Table S1

*Pearson and Spearman Correlations Between Personality and Episodic Memory*

| Trait | Episodic Memory | | | | | | | | |
| --- | --- | --- | --- | --- | --- | --- | --- | --- | --- |
|  | Combined | CERAD | | | Brave Man | | Logical | | |
|  |  | Immediate | Delayed | Recognition | Immediate | Delayed | Immediate | Delayed | Recognition |
| Neuroticism | -.08*/-.08* | -.07*/-.07* | -.05*/-.05* | -.05*/-.05* | -.08*/-.09* | -.03/-.04 | -.07*/-.08* | -.05*/-.05* | -.04/-.05 |
| Extraversion | .09*/.09* | .08*/.08* | .09*/.08* | .06*/.06* | .05*/.04 | .03/.03 | .08*/.08* | .06*/.06* | .06*/.06* |
| Openness | .20*/.19* | .17*/.17* | .16*/.16* | .08*/.08* | .13*/.10* | .11*/.11* | .18*/.17* | .16*/.15* | .16*/.15* |
| Agreeableness | .15*/.14* | .15*/.14* | .13*/.13* | .08*/.08* | .10*/.07* | .08*/.07* | .11*/.10* | .10*/.09* | .11*/.10* |
| Conscientiousness | .14*/.12* | .13*/.12* | .12*/.11* | .08*/.08* | .08*/.06* | .08*/.07* | .10*/.10* | .10*/10* | .08*/.06* |
| Sample size | 2817 | 2816 | 2803 | 2807 | 2807 | 2770 | 2790 | 2762 | 2756 |

*Note*. Coefficients are Pearson/Spearman correlations.

*p<.01.

Supplemental Table S2

*Pearson and Spearman Correlations Between Personality and Speed-Attention-Executive*

| Trait |  | Speed-Attention-Executive | | | | | | |
| --- | --- | --- | --- | --- | --- | --- | --- | --- |
|  |  | Combined |  | Letter Cancelation | Backwards Count | Symbol Digit | Trails A^a^ | Trails B^a^ |
| Neuroticism |  | -.08*/-.08* |  | -.03/-.05 | -.05*/-.06* | -.07*/-.07* | -.07*/-.06* | -.05/-.04 |
| Extraversion |  | .12*/.12* |  | .10*/.11* | .09*/.09* | .11*/.11* | .07*/.09* | .06*/.06* |
| Openness |  | .23*/.21* |  | .12*/.13* | .16*/.15* | .23*/.21* | .18*/.19* | .16*/.15* |
| Agreeableness |  | .16*/.14* |  | .10*/.10* | .11*/.10* | .13*/.12* | .11*/.12* | .10*/.09* |
| Conscientiousness |  | .19*/.18* |  | .12*/.13* | .12*/.12* | .18*/.17* | .14*/.14* | .15*/.16* |
| Sample size |  | 2808 |  | 2721 | 2786 | 2715 | 2736 | 2471 |

*Note*. Coefficients are Pearson/Spearman correlations.

*p<.01.

Supplemental Table S3

*Pearson and Spearman Correlations Between Personality and Visuospatial Ability, Fluency, and Numeric Reasoning*

| Trait |  | Visuospatial Ability | | | | |  | Fluency |  | Numeric Reasoning |
| --- | --- | --- | --- | --- | --- | --- | --- | --- | --- | --- |
|  |  | Combined |  | Constructional Praxis | | Pattern Reasoning |  |  |  |  |
|  |  |  |  | Immediate | Delayed | Raven Matrices |  | Semantic Fluency |  | Number Series |
| Neuroticism |  | -.09*/-.09* |  | -.06*/-.06* | -.08*/.08* | -.08*/-.09* |  | -.05*/-.06* |  | -.07*/-.07* |
| Extraversion |  | .06*/.04 |  | .03/.02 | .07*/.06* | .04/.02 |  | .09*/.09* |  | .00/.00 |
| Openness |  | .24*/.22* |  | .19*/.17* | .18*/.17* | .23*/.22* |  | .21*/.21* |  | .16*/.14* |
| Agreeableness |  | .11*/.08* |  | .08*/.05* | .10*/.08* | .09*/.05* |  | .12*/.11* |  | .02/.01 |
| Conscientiousness |  | .15*/.13* |  | .13*/.12* | .12*/.11* | .13*/.10* |  | .11*/.10* |  | .09*/.08* |
| Sample size |  | 2808 |  | 2796 | 2792 | 2791 |  | 2816 |  | 2457 |

*Note*. Coefficients are Pearson/Spearman correlations.

*p<.01.

Supplemental Table S4

*Adjusted R^2^ for each Regression Analysis Predicting Episodic Memory from Personality and the Covariates*

| Trait |  | Episodic Memory | | | | | | | | | | | |
| --- | --- | --- | --- | --- | --- | --- | --- | --- | --- | --- | --- | --- | --- |
|  |  | Combined |  | CERAD | | |  | Brave Man | |  | Logical | | |
|  |  |  |  | Immediate | Delayed | Recognition |  | Immediate | Delayed |  | Immediate | Delayed | Recognition |
| Neuroticism |  | .278/.287 |  | .169/.175 | .217/.223 | .092/.096 |  | .120/.127 | .108/.110 |  | .203/.221 | .208/.212 | .136/.137 |
| Extraversion |  | .278/.279 |  | .169/.170 | .217/.218 | .092/.092 |  | .120/.120 | .108/.108 |  | .203/.204 | .208/.208 | .136/.136 |
| Openness |  | .278/.283 |  | .169/.175 | .217/.221 | .092/.092 |  | .120/.121 | .108/.110 |  | .203/.208 | .208/.210 | .136/.139 |
| Agreeableness |  | .278/.282 |  | .169/.173 | .217/.219 | .092/.093 |  | .120/.121 | .108/.109 |  | .203/.205 | .208/.209 | .136/.139 |
| Conscientiousness |  | .278/.282 |  | .169/.174 | .217/.220 | .092/.094 |  | .120/.121 | .108/.110 |  | .203/.205 | .208/.209 | .136/.136 |
| Sample size |  | 2817 |  | 2816 | 2803 | 2807 |  | 2807 | 2770 |  | 2790 | 2762 | 2756 |

Supplemental Table S5

*Adjusted R^2^ for each Regression Analysis Predicting Speed-Attention-Executive from Personality and the Covariates*

| Trait |  | Speed-Attention-Executive | | | | | | |
| --- | --- | --- | --- | --- | --- | --- | --- | --- |
|  |  | Combined |  | Letter Cancelation | Backwards Count | Symbol Digit | Trails A^a^ | Trails B^a^ |
| Neuroticism |  | .391/.400 |  | .197/.203 | .260/.262 | .406/.413 | .228/.234 | .232/.238 |
| Extraversion |  | .391/.396 |  | .197/.201 | .260/.264 | .406/.409 | .228/.229 | .232/.233 |
| Openness |  | .391/.397 |  | .197/.201 | .260/.262 | .406/.411 | .228/.233 | .232/.235 |
| Agreeableness |  | .391/.400 |  | .197/.203 | .260/.267 | .406/.409 | .228/.233 | .232/.238 |
| Conscientiousness |  | .391/.406 |  | .197/.206 | .260/.265 | .406/.417 | .228/.236 | .232/.245 |
| Sample size |  | 2808 |  | 2721 | 2786 | 2715 | 2736 | 2471 |

Supplemental Table S6

*Adjusted R^2^ for each Regression Analysis Predicting Visuospatial Ability, Fluency, and Numeric Reasoning from Personality and the Covariates*

| Trait |  | Visuospatial Ability | | | | |  | Fluency |  | Numeric Reasoning |
| --- | --- | --- | --- | --- | --- | --- | --- | --- | --- | --- |
|  |  | Combined |  | Constructional Praxis | | Pattern Reasoning |  |  |  |  |
|  |  |  |  | Immediate | Delayed | Raven Matrices |  | Semantic Fluency |  | Number Series |
| Neuroticism |  | .313/.321 |  | .171/.173 | .179/.187 | .336/.342 |  | .205/.208 |  | .292/.297 |
| Extraversion |  | .313/.313 |  | .171/.170 | .179/.180 | .336/.336 |  | .205/.208 |  | .292/.292 |
| Openness |  | .313/.324 |  | .171/.178 | .179/.184 | .336/.345 |  | .205/.216 |  | .292/.294 |
| Agreeableness |  | .313/.318 |  | .171/.173 | .179/.183 | .336/.339 |  | .205/.211 |  | .292/.293 |
| Conscientiousness |  | .313/.321 |  | .171/.176 | .179/.185 | .336/.339 |  | .205/.208 |  | .292/.295 |
| Sample size |  | 2808 |  | 2796 | 2792 | 2791 |  | 2816 |  | 2457 |
